# From art to science: A bibliometric analysis of architectural scholarly production from 1980 to 2015

**DOI:** 10.1371/journal.pone.0276840

**Published:** 2022-11-03

**Authors:** Jean-Sébastien Sauvé, Philippe Mongeon, Vincent Larivière

**Affiliations:** 1 École de bibliothéconomie et des sciences de l’information, Université de Montréal, Montréal, Canada; 2 School of Information Management, Dalhousie University, Halifax, Canada; 3 Centre for Science and Technology Studies (CWTS), Leiden University, Netherlands; 4 Observatoire des Sciences et des Technologies (OTS), Centre Interuniversitaire de Recherche sur la Science et la Technologie (CIRST), Université du Québec à Montréal, Montréal, Canada; Universidad de las Palmas de Gran Canaria, SPAIN

## Abstract

According to recent literature on “architecture” as a discipline, practical knowledge relevant to its process of making has decreased in importance in favor of a more academic approach. Using data derived from Ulrich’s Periodical Directory and Clarivate Analytics’s Web of Science, this paper suggests providing empirical evidence supporting of such shift, as revealed by an overview of the dissemination practices in architecture scholarly production between 1980 and 2015. Our results support that architecture is becoming increasingly *academic*, as demonstrated by the growing proportion of articles and journals intended for scholars rather than for professionals. We also show that architecture is increasingly global, with decreased interest in local and/or national issues and the growing prevalence of English as a publication language. Finally, this academic focus is manifested in references cited by architectural papers with the gradual substitution of professional and artistic oriented knowledge, for scientific approaches tied to engineering and technology.

## 1. Introduction

Standing midway between arts and applied sciences, architecture is not easily definable unambiguously, as it encompasses both the abstract and the tangible. Architectural research combines several disciplines from arts (aesthetic judgment), engineering (materials, structures), practice (design, layout, and profession) and even social sciences (social and psychological receptions of space). While architecture, as an institutionalized academic discipline, needs to be distinguished from its associated professional activities, both technical-oriented architecture and academic architecture are taught in universities, influencing the nature of scholarly research.

The rupture between academic and technical knowledge stems from the absence of a consensus to define architecture as a field. In the introduction of the book *The Discipline of Architecture* [1], the editors state that the tension between professionals and scholars in architecture is due to the absence of a common vision and methodological approach. Along these lines, Robinson suggests in the essay *The Form and Structure of Architectural Discipline* that the opposition between architecture in professional studios and academic architecture results from the definition of architecture as a discipline. Robinson reports that architecture appeared initially in the United States in the 19^th^ century as an academic discipline, implicitly projecting a unified vision of the field [2]. This initial vision has led to architecture being considered as a discipline, along with other university departments, with its own teaching programs, research organizations and a wide range of publications venues, including specialized journals [2, 3]. As noticed by Robinson, however, whereas most people agree on the objects to be included in the architecture curriculum, there is no consensus on the topics’ denomination. More significantly from a research point of view, architecture is not recognized as a specific field by research funding agencies in the UK [4], the United States, the Netherlands [5] or Canada. In order to receive grants and research funding, researchers must therefore rationalize their programs within the scope prevailing in other related disciplines [6]. This lack of recognition could have arisen from architecture focusing on a single type of object (buildings) with no research methodology of its own and the need to borrow approaches from other fields.

According to Piotrowski and Robinson, a change in the paradigm is taking place in schools of architecture where “the need for specialization and more research has fed nonprofessional advanced education for which the studio may no longer be central, which creates a potentially expanding identity for architectural education beyond the professional orbit” [1]. Within the institutionalized context, the practical knowledge related to the process of making architecture decreases in importance, for a more academic approach with lesser attention being given to the professional aspects of the discipline. Architecture thus becomes an object of investigation shared by numerous approaches with multi- or interdisciplinary perspectives. Along this line, Deckker refers to a study of the British Academy underlining that “a recent review of institutions within the public (rather than university) academic and media worlds and their literature shows readily that architecture is no longer seen as a part of the humanities and social sciences” [7].

This observed shift in vision, in which architecture as a discipline favors academic over practical knowledge has a significant impact on institutionalized research production. Traditionally, technical knowledge expresses itself through a variety of drawings, models, etc., and can be considered as a language to understand an architectural solution or even as a communication plot [3]. The creation and experimentation of architectural designs, as practice-led research, are “unique as a subject and as a discipline” [4]. Practicing architects mostly present their work in professional journals and magazines that “feature photographs of built architecture rather than analysis of the buildings” [2].

Architectural research is mostly disseminated through the usual communication channels used in academia, such as monographs and scholarly journals [2]. It explores a wide variety of topics including the ideation of architectural objects, the objects themselves, the people associated with these objects, and the context within which these objects and people can be found. The development of research approaches that were initially limited to the tacit knowledge of buildings evolved to include “verbal evidence and justification for decisions in such forms as research studies, planning documents, cost-benefit analysis, and environmental impact analysis” [2].

In order to better understand the evolution of architectural research, this paper presents an overview of the research dissemination practices of architectural research between 1980 and 2015. We intend to determine if the shift in vision suggested by Piotrowski and Robinson, in which scholars substitute professional architecture for more academic knowledge production, is indeed taking place in the field of architecture. Three units of analysis are considered: journals, papers, and citations. The evolution in the number of journals will first be analyzed. Specific issues relative to the production of scholarly papers will next be addressed, including their evolution over time, geographic provenance, and language. Finally, collaboration and citation patterns will be investigated.

## 2. Methods

### 2.1 Bibliometrics

Bibliometrics uses scholarly publications to measure various aspects of research activity and scholarly impact [8, 9]. While its main application is in the context of research evaluation as a tool to provide quantitative assessments of research performance [10], bibliometric methods can also be used to understand researchers’ publication and collaboration practices, levels of interdisciplinarity and shifts in research topics. Bibliometrics thus can be used to provide empirical evidence of the alleged shift occurring in architecture schools proposed by Piotrowski and Robinson.

Architecture is generally considered to be part of the humanities. Research dissemination practices in those disciplines differ from those in the natural and medical sciences [11]. Gervits and Orcutt already underlined that quantifiable measures such as Impact Factors, citations and h-indexes are hardly applicable in the humanities—and especially in arts, architecture and design-related disciplines: “traditional tools for citation analysis are discipline-dependent; many are skewed towards science and technology, while art, architecture, and design which rely extensively on publications other than journals, are disadvantaged and deficient in their scholarly representations” [12]. Furthermore, in contrast to natural and medical sciences, recognition in the humanities is mostly obtained through the production of monographs [13, 14], which led certain authors to claim that monographs published in humanities “are like the main course of a meal [and that] journal articles and other scholarly communications are like tapas” [15]. Lastly, the objects of study are often national rather than international—as they generally are in the natural and medical sciences [13, 16].

### 2.2 Data

We retrieved all records with the serial types “Journal” or “Magazines” (strictly consumer and trade), and the subject “Architecture” from Ulrich’s Periodical Directory (Ulrich’s). This database compiles all journals created at the world level and therefore provides an indication of the rate at which new journals are created in the discipline of architecture. Three categories of journals have been considered [17]:

1. “Consumer”, which encompasses publications “such as magazines of general interest having an appeal to all audiences, special interest magazines with editorial content that appealed to readers, […] single interest magazines […], and to most literary magazines”;
2. “Trade”, which refers to the “business publications targeted to readers in […] employment or commerce: industrial or manufacturing; merchandizing or trade; institutional; and professional”; and
3. “Academic/Scholarly”, which corresponds to refereed journals identified through “a process combining publisher self-reporting about individual titles and independent Ulrich’s editorial research”.

A total of 1,915 records were recovered, of which 1,354 were kept after removing duplicates. The content types “Consumer”, “Trade”, “Academic/Scholarly” led to the retrieval of 257, 549 and 548 journal records respectively. This dataset provided bibliographical information on each journal, including editing country, founding and terminating (if applies) dates, audiences, and language. The aforementioned duplicates of magazines (due to national versions, translations or different formats like microfilms and digital) were removed from the data and renaming and/or fusion of journals and magazines were respected as far as possible.

We retrieved from Clarivate Analytics’ Web of Science (WoS) all articles published from 1980 to 2015 in the subject area “Architecture”. As the Web version of the WoS is not well suited for advanced bibliometric studies, the analyses presented in this work were based on WoS data provided by the *Observatoire des Sciences et des Technologies* (OST) in a relational database format. This database includes the three main WoS indexes, that is the Science Citation Index Expanded, the Social Science Citation Index and the Arts & Humanities Citation Index. In accordance with the criteria established in bibliometric analysis, specific publication types only were taken into consideration, namely research articles, notes, and reviews. A total of 71,448 articles published in 79 distinct journals were found. Although architecture is considered as an interdisciplinary field with important ties to arts, engineering, urban planning, etc., the Arts & Humanities Citation Index contains a specific subcategory labelled “Architecture”. According to Clarivate Analytics, this category “covers resources that are concerned with the study of art and science of buildings, particularly the design and construction of habitable structures”. It also includes: “resources on architectural history, landscape architecture as well as urban and country planning and design” [18].

As underlined by Van der Hoeven, WoS’s “Architecture” subcategory not only includes peer-reviewed academic journals but also trade and commercial journals such as *Architectural Digest* or *L’architecture d’aujourd’hui*. “The fact that they are included in the ISI suggests there are no rigorous transparent quality criteria in place that govern the ISI’s Art & Humanities Citation Index” [5]. Since journal classification may differ between WoS and Ulrich’s, the 79 architecture journals found in WoS were searched in the later to avoid duplicates; the number of articles included in our dataset was therefore reduced by 44, and the number of journals, by 6.

To observe the evolution of the architecture publications’ characteristics, we have divided the dataset in two segments for some analyses: 1980–1997 and 1998–2015. This division allows a better comparison between the first and second halves of the studied period.

### 2.3 Limitations

As noted by the French *Comité national d’évaluation de la recherche*, the WoS database has several limitations including a limited coverage, the exclusion of certain types of documents (such as books that are widespread in literature on architecture), changes in the journal titles, a classification of the journals by disciplines, non-discrimination of different individual authors with identical names, distribution of the work among authors without reflecting their proportional contribution and non-detection of negative or erroneous citations [9, 19, 20]. Furthermore, according to Sugimoto and Larivière, regional and national indexes–among which Brazilian, Russian, Chinese, and Korean–may appear in WoS, but the lack of data interoperability within the database causes an underrepresentation of these literatures [9]. As for architecture specifically, Van der Hoeven pointed out that it “has the poorest coverage in the ISI indexes of all disciplines represented in the university” [5]. Journal entries in the Ulrich’s database may also contain some inaccuracies: journal names may have changed throughout the years, others may have merged. The indexation of some journals may also be incomplete and not fully processed in the database [20]. Finally, since only citations to other Web of Science publications are included in our analysis, references to non-indexed materials such as books and patents are not included.

## 3. Results

### 3.1 Journals

**Fig 1:**
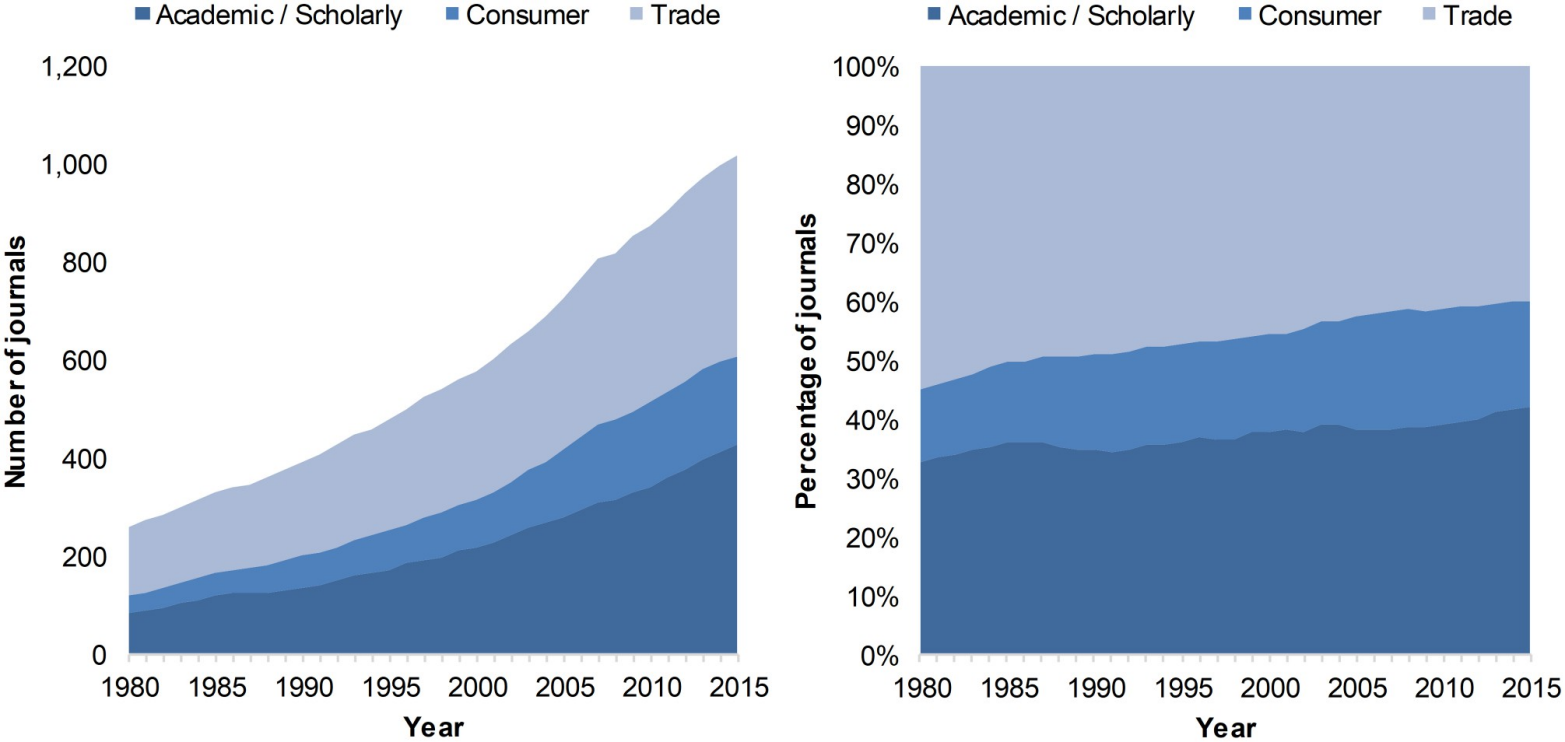
Number (left) and proportion (right) of scholarly journals devoted to architecture published worldwide, by year of founding and journal type, 1980–2015.

An analysis of the data retrieved from Ulrich’s shows that when considering only journals with a clear year of founding, the overall number of journals and magazines dedicated to architecture has increased between 1980 and 2015 (Fig 1, left panel). There was a small relative growth in academic/scholarly and consumer journal production over the period, while the proportion of trade journals slightly diminished (Fig 1, right panel). In absolute numbers, 376 academic/scholarly journals, 174 consumer magazines and 313 trade journals were launched, while 29 academic/scholarly journals, 24 consumer magazines, and 44 trade journals ceased their activities. Of note, academic and scholarly journals showed the most important net growth with 347 journals followed by trade journals (269), and consumer magazines (151).

### 3.2 Papers

**Fig 2:**
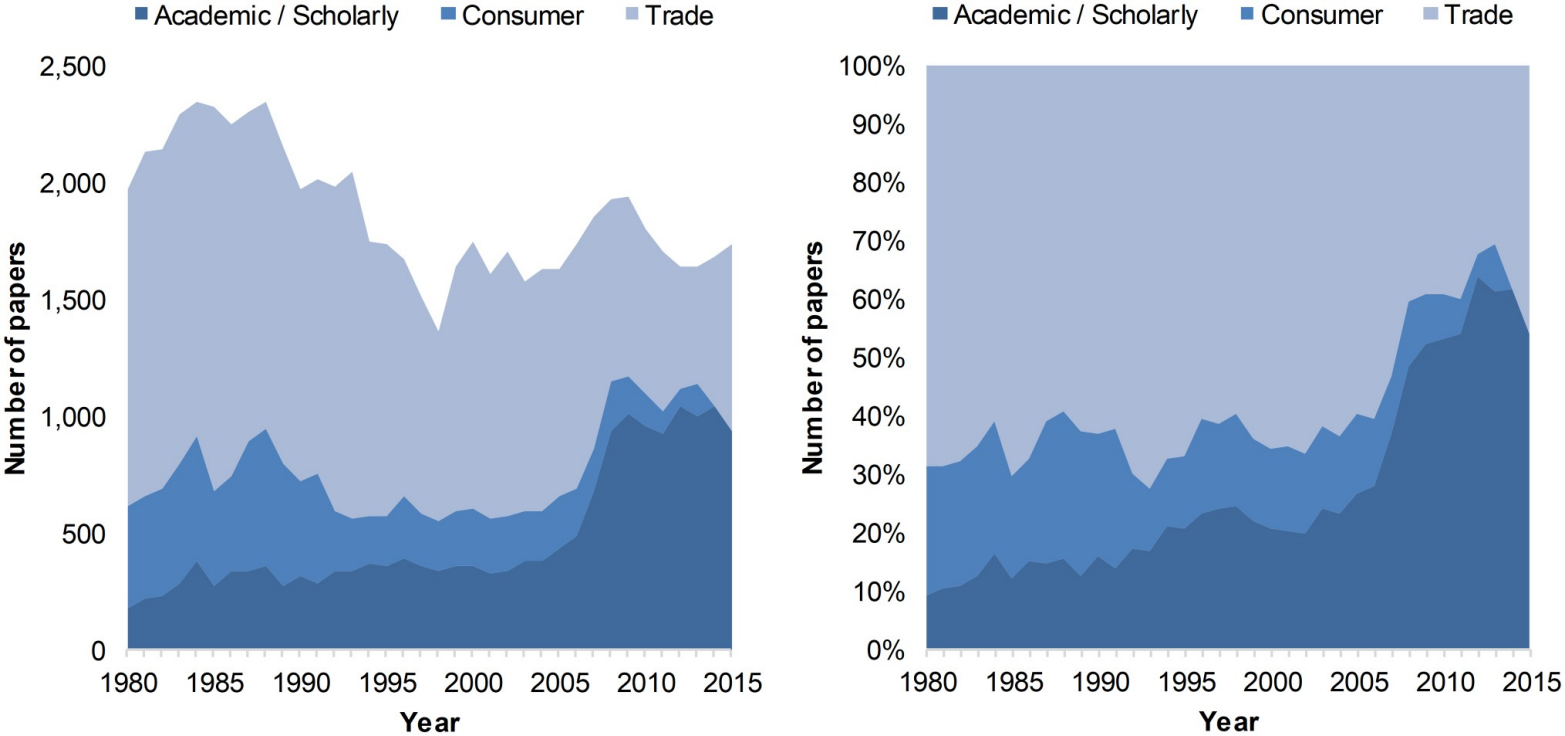
Number (left) and proportion (right) of papers devoted to architecture published worldwide, by year of publication and journal type, 1980–2015.

Despite the increased number of journals, the overall number of articles in the fields indexed in WoS slightly decreased from 1980 to 2015 (Fig 2). These results, obtained by classifying WoS journals according to their category in Ulrich’s, support the view that up to 2006 the total number of publications essentially reflects the evolution of trade publications. The increase in overall number of publications seen afterward correlates with the surge in academic/scholarly production that took place after 2006, with academic/scholarly papers becoming the dominant type of document. We also note that 1998 had the lowest output (1,362 articles), a result essentially reflecting a decrease in the number of articles published in trade journals and, to a lesser extent, consumer magazines.

The results presented in Fig 2 also indicate that the consumer category ranked second from 1980 to 1992 in terms of the number of papers published, before being outnumbered by articles in the academic/scholarly category. So despite a small increase from 1980 to 2015 in the number of journals in the customer and trade categories mentioned above, the number of articles indexed in the WoS diminished steadily over the same period.

A major feature of the results presented in Fig 2 (left panel) is the slow growth in the number of articles published in academic/scholarly journals. Articles in academic/scholarly journals outnumbered publications in trade journals in 2008 and remained since the category with the largest annual number of publications. This tendency becomes more apparent in Fig 2 (right panel), where the percentage of academic papers exceeds the fraction of papers from the two other categories (between 50% and 60% of overall journals). This may be partly related to the increasing number of academic/scholarly journals shown in Fig 1. It is also worth mentioning that consumption journals seem to disappear from our corpus in 2014 and 2015. One also has to keep in mind that the WoS indexing policy is likely to explain these trends, as this database mostly focuses on academic/scholarly publications rather than consumer and trade journals and magazines.

**Fig 3:**
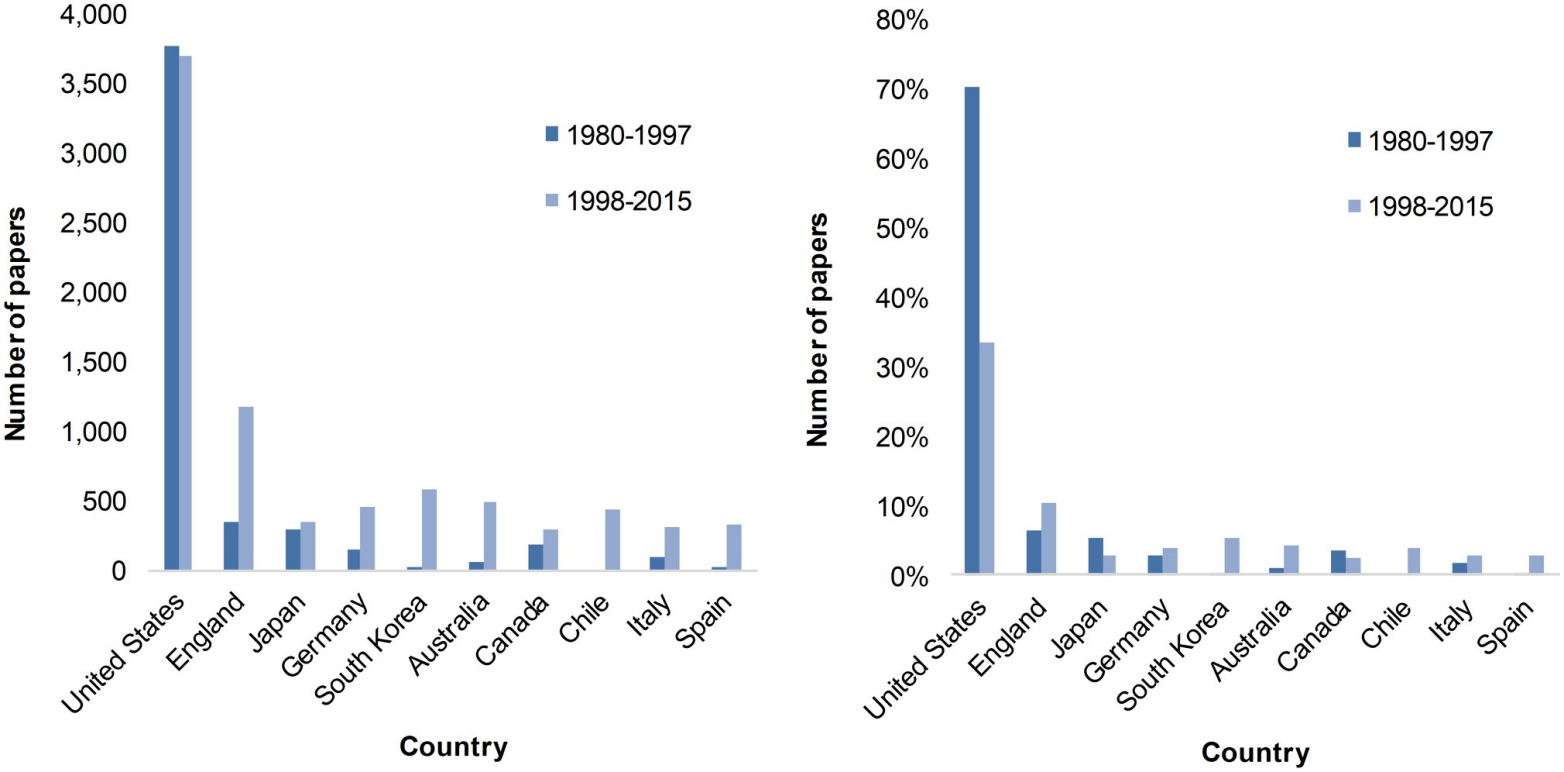
Number (left) and proportion (right) of scholarly/academic papers by country (top 10), 1980–1997 and 1998–2015.

The dominant place of US scholars in architecture papers is confirmed in Fig 3. We noted, however, that the percentage of scholarly/academic articles published by American scholars diminished drastically by more than half when comparing the 1980–1997 to the 1998–2015 periods. This was correlated by an increase in the second period of the number of scholarly articles coming from non-US countries, in particular England and South Korea, the latter increasing from 0.1% to 5.3%. The newly indexed journal *Space* in the WoS database during the second period may partly account for such a remarkable increase in South Korea’s publications. It is also worth noticing that, while scholars from the US (as well as English-speaking Canada) lose their prevalence at the international level, other English-speaking countries such as the United Kingdom and Australia are increasing their share of world papers.

Despite the decline of US papers, English remained dominant and grew in importance throughout the years (Table 1). In contrast, French, which represents the second most common language used, showed a sharp decline in the percentage from 14% to 4% over the same period. German and Spanish, that were not predominant before 1998, both gained publication space, whereas the use of Japanese and Italian decreased and can now be considered as marginal. Other languages (such as Hungarian, Czech, Finnish, etc.), which were not present initially, are still poorly represented with a total of 2.7%.

**Table 1:** Number and percentage of articles by language, 1980–1997 and 1998–2015.

Language	1980–1997		1998–2015	
	N	%	N	%
English	30,072	81.8%	26,756	88.7%
French	5,141	14.0%	1,364	4.5%
Spanish	0	0.0%	819	2.2%
German	201	0.5%	661	1.3%
Italian	622	1.7%	149	0.5%
Japanese	723	2.0%	15	0.0%
Others	0	0.0%	386	2.7%
Total	36,759	100%	30,150	100%

The results from this analysis should, however, be interpreted with caution as the Clarivate Analytics’ WoS tends to favor the English language, a bias already pointed out by many investigators. As a result, non-English-speaking authors, institutions, publishers, or countries are likely to be underrepresented [21]. Despite this bias, the data in Table 1 nevertheless provide clear indications of the dominance of English as the main publishing language in architecture—or at least in international journals—and that authors from non-English-speaking countries tend to publish more in English in journals often owned by American, British and Dutch publishers—that are overrepresented in WoS [21]—to participate in the global scientific dialogue.

### 3.3 Collaboration and citations

Troiani et al. observed that scholarly research in arts and humanities is “carried out by lone individuals, each acting independently, often raising questions rather than answering them, and, in architecture’s case, often testing architecture’s boundaries to ask what architecture might be” [4]. Such a culture seems to be confirmed by the data provided by WoS (Table 2). There is almost no collaborative authorship in scholarly journals during the 1980–1997 period with a total of 5,619 articles, of which 5,026 (89%) have a single author. However, a significant increase in the number of multiple author publications is seen during the second period, with 28% of all the papers published listing more than one author. The most collaborative article had 43 authors, a feature that is rather exceptional. Single authorship seems to remain the most current practice in architecture, even though it tends to become less prominent through the years. This is clearly at odds with natural sciences and engineering, where the majority of the scholarly papers list multiple authors [22, 23].

**Table 2:** Distribution of papers by the number of authors, 1980–1997 and 1998–2015.

Number of authors	1980–1997		1998–2015	
	N	%	N	%
1	5,026	89.4%	8,565	71.9%
2	478	8.5%	1,990	16.7%
3	83	1.5%	787	6.6%
4	12	0.2%	346	2.9%
5 or more	20	0.4%	218	1.8%
Total	5,619	100%	11,906	100%

In addition to mostly being the result of individual authors, publications in humanities, including architecture, tend to include fewer references compared to publications in other fields. Hammarfelt considers most humanities as a “rural field”, where there is low dependence on colleagues, less communication between the researchers, less publication channels, and thus fewer citations. Thus, “the social and intellectual organization of the humanities is the main reason to why citation-based approaches are less applicable in these fields” [14].

**Fig 4:**
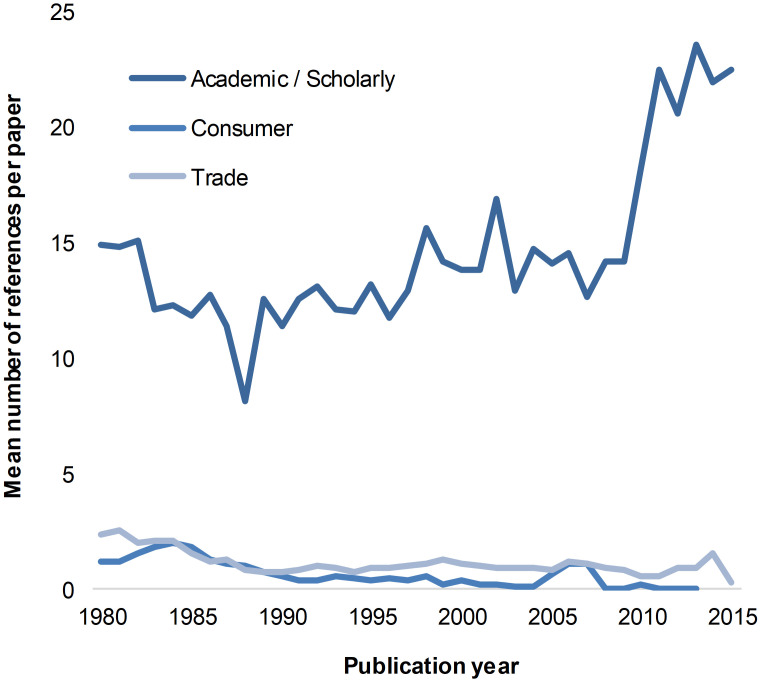
Average number of references per article, by year, 1980–2015.

Articles published in trade journals and in consumer magazines do not usually contain a reference list, as this is not standard practice in these types of publications. The data presented in Fig 4 indicates, however, that there was a noticeable growth in the average number of citations in academic/scholarly papers, and especially a rapid increase in the number of references per article from 2009 to 2015 (Fig 4). This correlates well with the data illustrated in Fig 2 where the number of articles published in scholarly journals drastically increased in 2007. Altogether these results support the general view that more articles were published every year, each containing more references and, therefore, more links to other scholarly literature.

**Fig 5:**
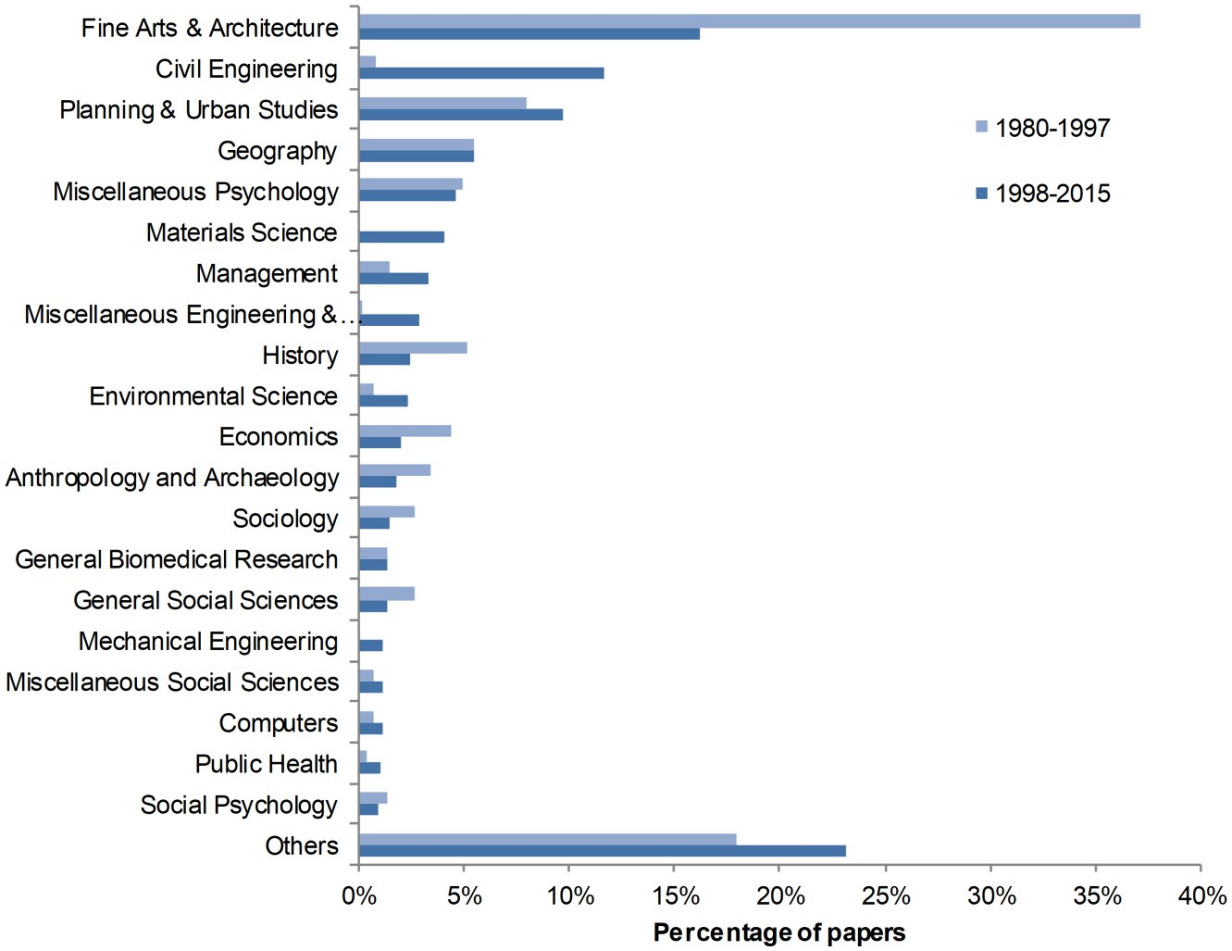
Proportion of cited references, by discipline, 1980–1997 and 1998–2015.

The disciplines to which scholarly papers in architecture are referring are presented in Fig 5. Fields are based on the journals in which the cited papers are published, and journals are assigned to one discipline of the National Science Foundation (NSF) classification. It shows that papers from our body of study are increasingly citing a wide array of disciplines. For instance, while papers in fine arts and architecture accounted for 37% of all cited references in 1980–1997, this percentage has decreased to 16% in 1998–2015. Various specialties of engineering are increasing over the period, such as civil engineering (from 1% of references to 12%) and material science (from less than 0.5% to 4.1%). While planning & urban studies are slightly increasing (from 8% to 9.7%), the share of reference to geography papers remains relatively stable over the period. Management and environmental science are both sizably increasing, although their proportion of cited remains below 4%. On the other hand, most fields of social sciences and humanities account of a lower percentage of references: history decreases from 5.2% to 2.5%, economics from 4.4% to 2.0%, anthropology and archaeology from 3.4% to 1.8% and sociology from 2.7% to 1.4%. On the whole, the data suggests that authors in architecture are increasingly citing more diverse disciplines, with a growing interest in engineering and technology and a decline in both social science and arts and humanities.

## 4. Discussion and conclusion

The data presented above suggests that publications on architecture are increasingly becoming academic. This is demonstrated by the growing proportion of articles and journals intended for a scholarly rather than professionals (trade), or grand public (consumer) audience. The overall number of journals and magazines dedicated to architecture belonging to the type scholarly/academic, trade, and consumer is growing every year testifying that their readership does not dramatically decrease. According to our body of study, academic/scholarly journals have, however, become the dominant type of publication in this field, outnumbering professional journals (trade) and the magazines intended for consumers. Trade and consumer papers seem to have lost their prevalent position and thus are relegated to a secondary position in the diffusion of architecture-related information, in favor of journals labelled as academic/scholarly.

The number of papers is not representative of the overall academic and scholarly production of articles in architecture as the WoS considers documents published in journals; which does not cover all of the communication channels used by the scholar community. In fact, it is well documented that authors in applied sciences, social sciences, behavioral sciences and humanities, tend to use other means of communication including books or multidisciplinary scholarly journals [9, 10, 24]. The trends in the number and characteristics of papers and journals may nevertheless express the transformations occurring within a given discipline as to answer to the new needs of the creators of knowledge and their readers. Our analysis has also revealed that the proportion of single-author papers has decreased; scholars in architecture appear therefore to be increasingly collaborative.

Although English is considered the *lingua franca* in sciences, national languages are more common in humanities due in part to the strong local or regional orientation of this field and the nature of the objects to be studied [14]. This being said, English plays now a predominant role in architecture scholarly publications, and, as observed in Germany by van Leeuwen, many scholars in humanities prefer to publish in English and not in their national language [25]. The choice of English over national languages tends to testify “a clear expression of an ambition to reach an international audience of experts in the field” [26]. So, despite an increasing contribution of new (and smaller) countries to the scientific dialogue, authors from both English-speaking and non-English-speaking countries globally choose to publish in English. This may reflect the globalization of questions revolving around architecture, and a decreased interest towards local and/or national questioning more characteristic in arts and humanities. One cannot also neglect the major role played by publishers based in the United States and in the United Kingdom, and the symbolic capital associated with these publishers, as a key determinant to explain the authors’ preference to publish in English.

The observed gradual growth in the overall number of references per paper may also suggest the academization of architectural research. Because architecture as a discipline does not have its own research methods, it must refer to other fields [3], thus explaining the need for scholarly publications to cite works coming from other disciplines—a phenomenon which is increasingly happening. References to arts and humanities and social sciences journals have decreased over the period 1980–2015, in favor of engineering and technology. Since it is assumed that authors are referencing papers which subjects are related to their own, this trend may express a gradual detachment from an artistic and humanities oriented knowledge towards scientific approaches emerging from engineering and technology and social sciences.

Our analysis attempts to provide empirical evidence of an academization of the field of architecture, thus confirming Piotrowski and Robinson’s observations [1]. However, the growing relationship between architecture and engineering and technology, as observed by the large increase of references to engineering journals, may suggest that the academization of the field does not arise from the development of the traditional culture in architecture based on arts and humanities and social science, but rather from a shift in the objects studied and the methodological approaches used. This academization is therefore not associated, as suggested by Piotrowski and Robinson, to a decreased importance given to technical knowledge compared to an academic type of knowledge production, but may rather be related to an increase in the relative interest in technical, technological and construction aspects of the field. Of course, scholarly publications are not the only witness of the vivacity and dynamism of architecture as a global field of knowledge: design and construction of buildings are also important achievements that reflect the progress and development of the discipline. Scholarly publications testify that architecture as a research field seems to be evolving to now become an academically technical discipline.
